# Supplementary material for: MetaRibo-Seq measures translation in microbiomes
Source: Nat Commun. 2020 Jun 29;11:3268. doi: 10.1038/s41467-020-17081-z (PMC7324362; doi:10.1038/s41467-020-17081-z)
Supplement: Supplementary file 10 — Supplementary Data 7 [file 41467_2020_17081_MOESM10_ESM.zip › File2/Confidence_High_Taxonomy/82128_out.krona.html]

Javascript must be enabled to view this page.

members
magnitude
magnitudeUnassigned
count
unassigned
taxon
rank

82128\_out

2

2
2
superkingdom

phylum
1
2
1239

SRS893383\_contig\_number\_contig-100\_6181.6182

1
class
186801

186802
order
1

1
family
186803

1898203

SRS019910\_contig\_number\_contig-100\_21463.78669
species
1
